# Professional Use of Social Media by Pharmacists: A Qualitative Study

**DOI:** 10.2196/jmir.5702

**Published:** 2016-09-23

**Authors:** Arcelio Benetoli, Timothy Frank Chen, Marion Schaefer, Betty B Chaar, Parisa Aslani

**Affiliations:** ^1^ Faculty of Pharmacy, The University of Sydney Sydney Australia; ^2^ Department of Pharmaceutical Sciences, State University of Ponta Grossa Parana Brazil; ^3^ Charité University Medicine Berlin Berlin Germany

**Keywords:** social media, social networks, social networking sites, YouTube, Wikipedia, Facebook, pharmacists, pharmacy

## Abstract

**Background:**

Social media is frequently used by consumers and health care professionals; however, our knowledge about its use in a professional capacity by pharmacists is limited.

**Objective:**

Our aim was to investigate the professional use of social media by pharmacists.

**Methods:**

In-depth semistructured interviews were conducted with practicing pharmacists (N=31) from nine countries. Interviews were recorded, transcribed verbatim, and thematically analyzed.

**Results:**

Wikipedia, YouTube, and Facebook were the main social media platforms used. Professional use of social media included networking with peers, discussion of health and professional topics, accessing and sharing health and professional information, job searching, and professional promotion. Wikipedia was the participants’ first choice when seeking information about unfamiliar topics, or topics that were difficult to search for. Very few pharmacy-related contributions to Wikipedia were reported. YouTube, a video-sharing platform, was used for self-education. University lectures, “how-to” footage, and professionally made videos were commonly watched. No professional contribution was made to YouTube. Facebook, a general social networking site, was used for professional networking, promotion of achievements, and job advertisements. It also afforded engagement in professional discussions and information sharing among peers.

**Conclusions:**

Participants used social media in a professional capacity, specifically for accessing and sharing health and professional information among peers. Pharmacists, as medicines experts, should take a leading role in contributing to health information dissemination in these user-friendly virtual environments, to reach not only other health care professionals but also health consumers.

## Introduction

Social media (SM) encompasses a wide range of websites whose content is created by users [1]. While the Internet has increased public access to all kinds of information, its evolution to Web 2.0 has provided a more participatory environment where users not only access, but also create, edit, and share content online in several formats (eg, text, picture, audio, and video). SM has transformed communication, including health care communication [2]. Consequently, SM is increasingly shaping the way health care professionals work and provide their services. The Internet can be considered as an important tool for pharmacists and other health care professionals for their daily professional activities, particularly as a medium to access health information [3]. It is believed that SM has further impacted the way health care professionals deliver their services by increasing their knowledge, efficiency, communication with patients, marketing, and communication with colleagues [4]. However, the influence of social media use by pharmacists in a professional capacity has not been fully investigated, with limited literature on the use of social media by pharmacists [5].

Studies conducted to date have focused on specific SM platforms such as Wikipedia [6], blogs [7,8], social networking sites (SNS) [9,10], and Twitter [11], with only one study addressing SM more broadly, (ie, including several different types of platforms) [12]. One study has shown that in 2009, 35% of US pharmacists were using Wikipedia for general purposes and 10% for drug information [6], while another study found that 72% of pharmacists surveyed when attending a regional pharmacy conference in the United States used Wikipedia [12]. Neither study, however, investigated how Wikipedia supported pharmacists’ work or their perceptions of its usefulness. In terms of blogging, 44 [8] and 136 [7] pharmacists’ blogs were identified and analyzed in two separate studies published in late 2010. These blogs provided information about news and current events in health care, pharmacological discussions, and pharmacists’ personal views on their professional and private lives. To date, pharmacists’ professional use of SNS appears to be incipient, with personal and social intentions being the main motivations for use [9,10,12]. For instance, 90% of pharmacy preceptors in a US survey reported using Facebook primarily for personal and social reasons [9], and 90% of pharmacists’ tweets were predominantly or exclusively for personal purposes [11]. Pharmacists’ Facebook use ranged from 50% to 67% [9,10,12]. Its use was more common among younger practitioners (<29 years old), decreasing as age increased [10]. At the time of the study, the Twitter user rate among pharmacists was very low [9,11], with the most recent study revealing that less than 1% of US pharmacists were active on Twitter [11].

Although some research about the use of SM platforms by pharmacists has been conducted, these studies have either used a quantitative survey [6,9,10,12,13] or a direct observation of SM platform approach [7,8,11] with little in-depth exploration of SM use. Therefore, the current study aimed to build on previous research by using a qualitative approach to investigate how pharmacists perceive and use SM professionally.

## Methods

### Qualitative Methodology

This was an exploratory qualitative study that employed in-depth, semistructured interviews to explore pharmacists’ use, opinions, and perceptions of SM. A qualitative approach was considered most suitable given that our aim was to elicit rich and detailed aspects of pharmacists’ professional use of SM and explore their perceptions and opinions about it. Another advantage is that qualitative research allows the investigators to explore the viewpoints of individuals in detail, with a small number of participants [14]. Ethics approval was obtained from the University of Sydney Ethics Committee (Project No. 2013/635, approval date: 14/08/2013) prior to commencement of the study.

### Participants and Recruitment

This study sought a global perspective from pharmacists’ use of SM, so a purposive sampling was used to recruit pharmacists from a range of countries. Initially pharmacists known to the research team were invited, and thereafter participants were recruited using a snowballing technique. This strategy allowed the research team to take advantage of its international professional network (the research team comprised individuals from different countries: Australia, Brazil, and Germany) and identify active SM users within the pharmacy profession with a range of professional roles. Pharmacists from different professional settings and countries took part in this study. All participants received a participant information statement and verbal explanation about the project’s aim, the research team and the interviewer, and their participation prior to the interviews. Consent was acquired by having participant sign a standardized consent form.

### Data Collection

Semistructured interviews were conducted between November 2013 and December 2014. An interview guide (Multimedia Appendix 1) based on the study aim and objectives was developed and piloted with 5 participants to assess face and content validity. The piloted data were not included in the analysis. The interview guide consisted of topics related to participant understanding of SM, SM usage, and perception of SM use for health care and in the pharmacy profession. All questions were open-ended to allow relevant topics and themes to emerge without constraint. Whenever needed, follow-up questions were asked in order to clarify or expand participants’ comments. The interviews were conducted either face-to-face, by telephone, or Skype (voice call or video call), and audio-recorded with the consent of participants. Notes were taken to assist the formulation of prompt questions and support data familiarization for data analysis. The interviews were conducted in dedicated premises at the Faculty of Pharmacy, University of Sydney; they lasted from 30 to 130 minutes and were all conducted by AB, a male pharmacist and PhD candidate trained in qualitative research. Interviews were conducted until saturation was reached.

### Data Analysis

All interview recordings were de-identified and transcribed verbatim. One participant asked to see their transcript, but no revision or correction was made. A thematic analysis with an inductive approach was employed to identify themes within the interviews’ transcripts. Thematic analysis is not aligned with a particular epistemological, philosophical, or theoretical approach and is a flexible tool to generate themes in qualitative analysis [15]. Besides being a robust and sophisticated research tool, thematic analysis focuses and presents the data in a way that is readily accessible to those who are not part of academic communities [16], which benefits the research findings’ dissemination among pharmacy practitioners.

Repeated reading of transcriptions allowed familiarity with the data and knowledge of each interview’s content depth and breadth. After immersion within the data, transcriptions were coded line-by-line and collated within each code with the help of the software NVivo 10 (QSR International). The limited literature available on the topic [5] associated with the adoption of an inductive approach [17] ensured a data-driven analysis. Therefore, the coding process was open, not restricted by theoretical assumptions from the research team. The coding process was dynamic, iterative, and evolved throughout the analysis. Codes with a repeated pattern across the data (ie, codes with similar or nearly similar meanings) were grouped into subthemes and later assembled into overarching themes. Themes were carefully named according to their overall content. The first three interviews were separately coded by 2 researchers, and the coding labels and identified themes and subthemes were thoroughly discussed and agreed upon. Throughout the analysis, the coding process, including its grouping into themes, was discussed with a senior member of the research team (PA).

## Results

In total, 31 pharmacists from nine countries were interviewed (Table 1). Only one participant approached via email did not take part. Most participants were practicing in community pharmacy and academia (in the field of pharmacy practice).

**Table 1:** Participant demographics.

Demographics		n
**Gender**		
	Female	16
	Male	15
**Years of practice**		
	Range	2-37
	Median (SD)	13.3 (9.83)
**Work setting**		
	Community	14
	Academia	11
	Community & academia	2
	Pharmacy/health organization/industry	4
**Country**		
	Australia	17
	New Zealand	5
	United States	2
	Brazil	2
	Germany	1
	Nigeria	1
	Thailand	1
	Philippines	1
	United Kingdom	1
**Type of interview**		
	Face-to-face	10
	Telephone	8
	Skype	13

The first aspect explored in the interviews was pharmacists’ understanding of SM, and subsequently the interview focus shifted to actual use of these platforms with a focus on professional aspects.

### Pharmacists’ Knowledge and Understanding of Social Media

**Figure 1:**
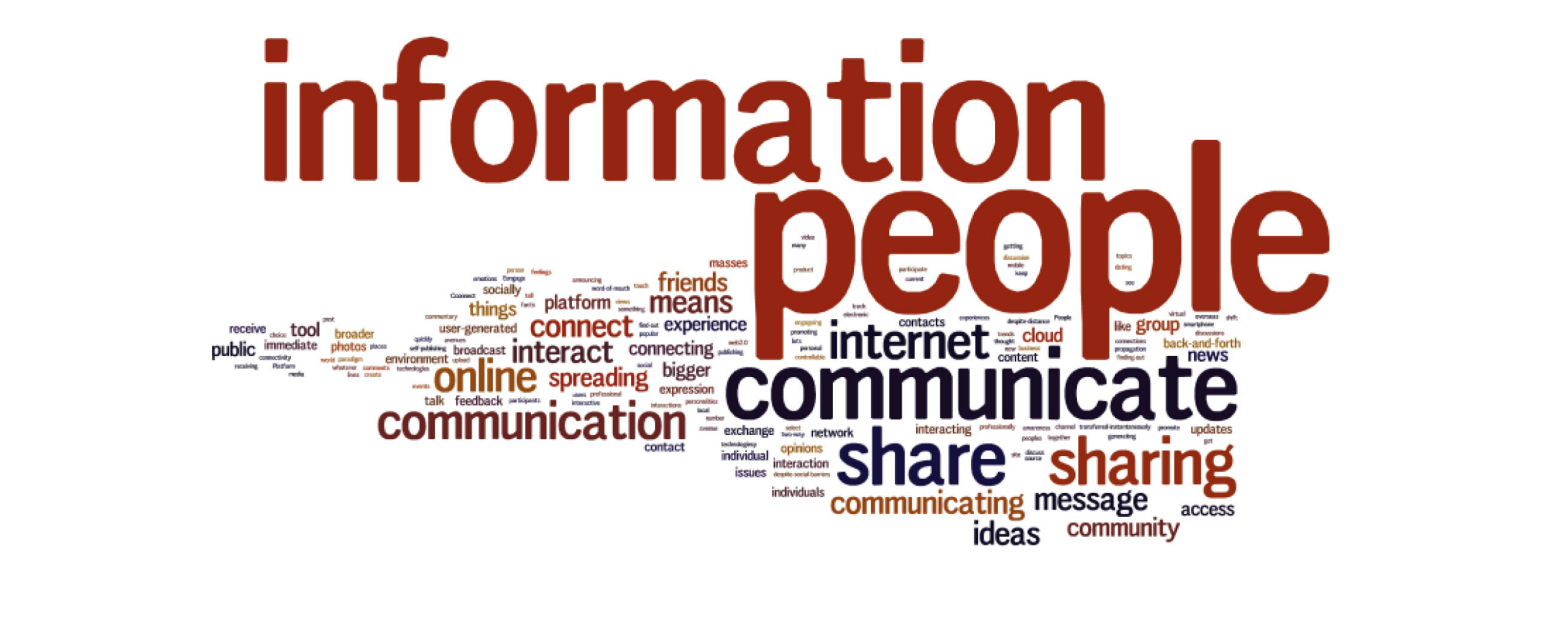
Word cloud of participants’ understanding of social media.

Most participants considered SM to be an online (Internet) community venue where information is accessed and shared with the aim of fostering communication. Figure 1 illustrates the concepts that participants associated with SM and portrays a broad description of their understanding. The word cloud was created using the key words (nouns and verbs) participants used when describing SM.

SM was considered as a new way to communicate and keep in touch with a large group of people publicly. Participants believed that this interactivity created a connection among users and allowed people to keep a virtual network of contacts, including a professional network.

As a source of information, SM was perceived to have the advantage that the users had a high level of control, allowing them to choose the content to read, listen, and watch. Some participants expressed that the information shared on SM platforms was created by the users themselves. However, only a few participants could explain the user-generated content aspect of SM: “It’s most like a forum for user-generated content. It’s the opposite of maybe company-generated websites; you’ve got the people themselves generating content and sharing it, communicating about it” [Participant 21].

SM interactivity was a feature highlighted by several participants: “You interact with those who posted the information…you can question. It’s a quick way to get in touch, with questions and answer. You hadn’t such a quick and effective interaction before Facebook” [P24].

Participants were also asked to provide SM examples. This permitted further evaluation of participants’ understanding of SM, through a comparison of the examples provided and the definition of SM given. Facebook was cited by all participants as an example and most times was the first example that “sprang to mind.” Popular SM platforms without SNS features (eg, public profile and list of contacts) like YouTube and Wikipedia were much less frequently cited. Twitter, although a microblog, possesses some SNS features and was commonly mentioned. Other common SM examples were LinkedIn, Instagram, Google Plus, and blogs. Virtual worlds, like Second Life, were rarely mentioned.

Would you consider Research Gate (a professional social networking site for academics, researchers, and scientists) social media?” [P10] and “I have a doubt. Is Moodle (a free software whose purpose is to serve as a learning platform via creation of personalized learning environments [18]) considered a social media platform?” [P23]

After obtaining participants’ definitions of SM, including examples, the SM operational definition (ie, a group of interactive platforms via which individuals and communities share, co-create, discuss, and modify user-generated content employing mobile and Web-based technologies [19]) was provided to ensure that everyone had the same understanding before continuing with the interviews.

### Pharmacists’ Use of Social Media Platforms

This theme details how pharmacists used or perceived SM use by their peers in a professional capacity. All participants reported using SM in a professional capacity, though to varying degrees. The most common platforms were Facebook, Wikipedia, and YouTube. Pharmacists used SM to network with fellow pharmacists, to access and share professional news and health- and medicine-related information, to discuss relevant topics, to exert political influence within the pharmacy profession, to promote their careers, and to find or assist peers to get employment. SM was also used to promote pharmacy stores and in the education of pharmacy students.

#### Use of Social Networking Sites

The use of SNS ranged from personal to professional. Facebook was the most used SNS platform. Although the majority of participants initially stated that their use of SM, particularly SNS, had a personal and social aim, many professional activities were identified during the interviews. In fact, after articulating their use of SM throughout the interview, some participants realized that their initial statement about SM use was not completely accurate. Some online activities were later categorized as professional ones. Interestingly, some participants were surprised to realize they had an extensive professional use of social media, even though not directly related to patient care.

Most participants reported using only one SNS regularly, Facebook, despite having accounts or profiles on other SNS (eg, LinkedIn, Twitter). Facebook was preferred due to its popularity (many users), simplicity (easy to use), and versatility (great range of features available). Facebook was regarded as a convenient venue, a “one stop shop” [P13], allowing the convergence or integration of different SM platforms.

Some accessed Facebook multiple times a day, using mobile devices (eg, smartphones, tablets). Differently from academia and research, SNS use was commonly restricted during work time in community pharmacies. A few participants reported that the dispensing computers had the Facebook webpage blocked, or even the use of their personal mobile phones was totally restricted: “In my pharmacy, we have a computer. We have guidelines for the usage of computer and Facebook is blocked in the pharmacy that I work at” [P17] and “So even your mobile phone shouldn’t be on you. Your mobile phone is locked in the staffroom and you don’t have access to your mobile phone while you’re working” [P2].

Personal motivations, namely communication and interaction with family and friends, were the main driving force for participants to join Facebook. Facebook was regarded as an inexpensive, quick, and easy way to connect with friends and family, including those overseas. As used primarily for personal reasons, participants were much more engaged with Facebook than with other dedicated professional platforms. However, eventually they started using Facebook professionally. Networking with colleagues on SNS was perceived to be an important professional interaction allowing some participants to break the isolation they felt, especially those working in regional or rural areas.

### Social Media for Learning and Information Sharing

SM platforms were used as professional learning channels for pharmacists. Facebook served as a convenient vehicle to easily access professional and health-related information from pharmacy organizations and peers. Following pharmacy organizations’ Facebook pages to receive professional updates and information on their news feed was commonly reported by participants: “I find it’s the quickest way of knowing what’s going on rather than me going to their [organizations’] website or finding out by email and taking the time to read the email. I think it is much quicker” [P10].

Participants were also active in sharing information. A variety of sources such as health authorities, health providers, pharmacy organizations, and traditional media (eg, newspaper articles and television reports) were used. Pharmacists described being engaged in posting information, often providing the primary source of the information: “It [the news] can be from a TV channel website, newspaper, magazine… but I always provide the link to the source of information” [P24] and “I believe we are able to understand the primary source of information, so I find information from those sources and I deliver that to my [group] members” [P31].

With regard to Twitter usage, participants either did not use it, or they heavily used it for professional purposes. Those who were tweeting did so in order to receive, provide, and share professional news, raise awareness about pharmacy issues and pharmacy services development, and/or promote their organization or institution. Twitter was also used as a search engine for certain trending topics, particularly for more abstract keywords/concepts or terms not well-established, using the hash tag and the keyword:

If I’m interested in a particular idea or topic I’ll search a #tag for whatever I’m interested in…I might be able to search for something a bit more abstract…I haven’t quite thought about why I would use specifically Twitter over Google but I think if the word that I’m looking, the key words are a bit more abstract, Google doesn’t really pick them up necessarily. Twitter might pick up those words better because they’re so generic. Google doesn’t know what exactly I’m looking for, but the way other people use that word like when they make a tweet, it’s very specific

P5

Twitter was also the preferred platform during conferences, to increase conference visibility, reach non-attendees, and increase delegate participation.

Wikipedia was the only wiki mentioned and reported as a commonly sought source of information, frequently accessed during working hours. Wikipedia was regarded as a convenient initial resource for searching for information because it provided background knowledge for further reading and learning: “If I’m looking for something I know nothing about, that can be a really good starting point” [P17].

Additionally, Wikipedia was also used when well-established common references failed to provide the information needed, and then Wikipedia references were commonly scrutinized in order to expand the information search.

It appeared that participants had their own rules about when it would be appropriate to use Wikipedia in a professional capacity. For instance, it was not used as a reference source to guide their professional actions. However, it was used when a general knowledge about a topic or quick access to information was required. Pharmacy-related information retrieved ranged from brand names, to pharmacological effects, to therapeutic classification and uses of a drug or medicine, to unfamiliar health topics, such as unfamiliar medicines or rare disease states.

Several characteristics of Wikipedia made it a useful SM tool for the participants. These included the speed with which participants could obtain information, knowing the date of the last update, the fact that entries were referenced, its not-for-profit nature, and usability. Despite the frequent use of Wikipedia by most participants, it was not regarded highly by many. Participants generally provided some rationale for not considering Wikipedia as a reputable source of information, such as lack of credentials and anonymity of Wikipedia contributors, and inaccuracy of some information. Therefore, the academic participants were not eager to recommend Wikipedia as a resource/reference for student use. Only 2 participants were engaged in editing Wikipedia entries, though just one contributed to medicine- and pharmacy-related information.

Four participants had their own blogs. Each of them had different purposes and audiences. They were used for pharmacy education (detailed below), entertainment (to publish drawings and cartoons created by the blogger), rural and aboriginal health care, and pharmacy profession in general. All those actively blogging emphasized that blog traffic generally depended on links to them being spread on SNS: “When I post on a blog, I promote that onto Twitter and Facebook” [P26] and:

Depending on the news significance, after I publish that on the blog I will provide a link to it on Facebook…most access to the blog comes from Facebook...when I publish a post on Facebook with a link to the blog, that news is much more accessed than other news not published on my Facebook account

P24

Though very few participants mentioned accessing blogs, the pharmacist hosting the general pharmacy blog reported more than 850,000 visits during 2 years of the existence of the blog, with an average daily audience of 1700 in 2014. Topics published in the pharmacy blog included job opportunities (both private and public sectors), pharmacy and pathology laboratories for sale, pharmacy fixtures and fittings for sale, professional events (eg, talks, workshops, and conferences), pharmacy legislation changes and updates, product recalls, new drug and treatment studies, and disease outbreaks (eg, Ebola). The most popular topics were those related to daily pharmacists’ working lives (eg, legislation affecting work procedures, community recognition, and level of trust in the profession). However, the most popular topic so far was a product recall in that country, with more than 23,000 visits.

The most used video sharing platform was YouTube. The video sharing site Vimeo was mentioned by a few participants, but its use was not common. Even though entertainment was the major motivation to access YouTube (eg, watching music clips), some professional uses were reported, such as obtaining content on technical information. Some participants expressed a preference for watching a video instead of reading, especially when dealing with practical procedures and “how-to-do things”: “Every now and again if I forget how to use a technique and need help, go back to YouTube, usually it’s from a professional organization, but it’s a YouTube link anyway” [P31]. Professionally made videos and lectures from reputable universities and educational institutions were highly used as self-learning material.

Some participants used YouTube frequently and considered it a good repository, while others were not regular users and did not access any professional-related content. None of the participants uploaded professionally related videos on social media platforms.

### Professional Discussions

SM provided an alternative venue for professional discussion and peer collaboration. Discussions on SNS, particularly Facebook and Twitter, were preferred over pharmacy blogs. Pharmacy- or pharmacist-themed private groups on SNS were very common for this purpose. The “Facebook groups” function allows users to create or join groups based on common interests [20]. These professional pharmacy groups were set up either by pharmacy organizations, such as the Pharmaceutical Society of Australia, or individual pharmacists. Membership in these groups was restricted to pharmacists, and the groups functioned as forums where discussion about regulations, provision of services, and professional ethical issues took place. The ability to provide and obtain information quickly was an important driving force in these groups. However, not all professional Facebook groups were constantly active, and activity could range from daily to quarterly. Moreover, within each group, individual members’ participation varied from a predominantly observational role to active participation.

Those who contributed more to the pharmacy Facebook forums were perceived to be the ones with stronger opinions. Thus, the moderation task was regarded as crucial.

### Pharmacy Jobs, Career, and Social Media

Participants believed that SM had an impact on access to the pharmacy job market by increasing visibility of both job opportunities available and prospective employees’ resumes. In terms of recruitment, although no participant mentioned using SM for their own benefit, it (namely pharmacy-themed closed Facebook groups and pharmacy blogs) was widely recognized as a medium for both employers and employees within the profession: “Job advertisements get utilized a lot, so on a local [Facebook] pharmacist’s page a lot of people will post up ‘job needed’ or whether they are looking for a job or whether they are trying to fill that job, so that’s quite common” [P31].

Although several participants had LinkedIn profiles to convey their professional qualifications and achievements, the vast majority of participants were not actively engaged with it. Its main purpose, as perceived by participants, was to increase professional visibility among peers and potential employers and be used for job searching purposes. Professional achievements were also posted on other social media outlets, particularly Facebook: “I know that there are colleagues that use Facebook to disseminate their research data so if a publication comes up they’ll put on their status update” [P15].

Additionally, some pharmacy practice academics reported having an account on Research Gate, a kind of “Facebook for science” [21]. The reported advantage of this social network was the formation of a clear professional network, the opportunity to promote one’s own research (eg, uploading articles), and establishing a network of researchers within their field. Pharmacy academics were aware that their research papers could be accessed by other researchers without access to expensive databases. However, none expressed a great level of activity or enthusiasm about its use.

### Social Media Use in Pharmacy Education

Social media also had its place in the delivery of pharmacy education. It was perceived to facilitate the learning experience in the current pharmacy student population that uses these platforms extensively and routinely for most aspects of their lives. Most pharmacy academic participants reported using SM for teaching purposes, though at different intensity and frequency. The teaching activities using SM were performed in class or designed to supplement in-class activities and served as an alternative method for the usual education management systems (eg, Moodle, Blackboard) used by pharmacy schools.

The major platforms used were Facebook and YouTube. It was recognized that information posted on Facebook would reach students faster because they perceived Facebook as the students’ favorite way of accessing course information as they were always accessing their personal Facebook pages.

Some of the pharmacy academic participants could be identified as early adopters of technologies. They tended to provide more examples of SM use in their pharmacy teaching. For these early adopters, SM was seen as an integral part of teaching and they tended to use different types of SNS platforms and groups for different teaching-related activities. Although the use of Facebook was perceived to be beneficial to supplement in-class activities and to improve both academic-student and student-student communication, its use within the classroom without teacher’s guidance was not regarded advantageous since it disrupted student concentration, preventing them from paying due attention or engaging in the topic being taught and discussed.

YouTube was perceived as a good resource to better illustrate concepts during lectures, provide supplementary information, serve as alternative learning resources, and increase student understanding especially students who were more visual learners. Wiki platforms were also used, specifically to assign group tasks to students, enabling the educators to gain a more accurate understanding of individual student participation. This “virtual control” was regarded as one of the great advantages of wiki platforms for educational purposes.

Even though pharmacists were using SM websites for self-education and educating students, they were not using it to educate other health care professionals. One interesting exception was the provision of a blog as a reference on how to write prescriptions, which was regularly recommended by a participant to junior physicians in a hospital.

### Other Uses of Social Media

Some of the academic participants had also used SM for research, specifically for recruiting participants or collaborators to research projects. SM was also used to collect data through posting questionnaires on Facebook.

As SM is a two-way platform, some participants used it as a medium for sparking discussions about the political issues within the profession and health sector, as well as voicing opinions about changes in professional activities. Consequently, Facebook also served as a venue to put pressure on decision makers and policy makers either for change or to demand more accountability from the profession’s leaders:

I think what’s important is to provide an avenue where pharmacist can use it as an outlet for some of their I guess frustrations with policy decision makers...Will it lead to change? I think it just really depends on the numbers...I think it will also force them to respond in a manner…to justify the decisions that have been made simply because of the reaction, the outbursts on these social websites

P18

While the major aim of this project was to explore how pharmacists used and perceived peers’ use of SM from a practitioner’s perspective, some additional relevant information on how SM is used by pharmacy stores was also identified. Many community pharmacies were reported to host a Facebook page, with a marketing aim. Most frequently, the Facebook page served as a supplementary or additional form of promotion about products already advertised (eg, in the pharmacies’ catalogue), as well as services available. Pharmacy Facebook pages were also reportedly used as a channel to spread general health information to the community.

## Discussion

### Principal Findings

This paper provides the first insight into the broad professional use of social media by pharmacists. It was believed that SM usage in pharmacy ranged from only social and personal communication (eg, with friends and family) at one end of the spectrum, to professional communication only (eg, with colleagues and clients) at the other end of the spectrum [22]. However, our study has shown that the professional use of SM by pharmacists is common and very diverse. Although many participants initially believed that their use of SM was exclusively for personal reasons, it became apparent during interviews that all participants used social media in a professional capacity. Even those few participants without an SNS account commonly accessed popular social media websites like Wikipedia and YouTube for professional purposes. The lack of awareness of professional use of SM by the participants could be attributed to how social media was initially narrowly defined and perceived.

Peer communication was one of the most common professional activities on social media. Participants preferred to use general SNS, like Facebook and Twitter, instead of pharmacy-only SNS for professional networking (eg, PharmQD). Similarly the use of professional SNS (eg, LinkedIn) was far less common. As reported in a separate publication [23], most participants had a blended approach [24] when using SNS, which means they had both professional and personal activities taking place in the same SNS. Participants’ initial motivation to set up a Facebook profile was social and personal in nature, and over time gradually evolved to have a mixed audience (both social and professional contacts) as pharmacy school friends became professional peers and colleagues became closer friends. Not surprisingly, professional online conversations and discussions then took place over a platform initially intended for personal reasons, like Facebook. Since community pharmacists commonly do not work alongside peers [25,26], this increased interaction among pharmacists provided by SNS would be a major advantage because it could diminish pharmacists’ isolation helping them to better deal with technical, clinical, and ethical issues that arise at work. However, this increased interaction is not taking place yet during pharmacists’ time in the community pharmacy as the use of these platforms was commonly restricted.

A natural development that followed the increased professional interaction afforded by SNS was the increased access and further spread of relevant professional information. In fact, one of the major professional activities performed by pharmacists on SM was to access information. It is widely known that health care professionals frequently use the Internet as a working tool, especially to access information [3]. As the Internet has shifted to be more interactive [1], it would be expected to find pharmacists using SM as an information source for work purposes and as a tool to learn from a variety of sources, such as from organizations, peers, and patients. So obtaining health and medicines information via SM platforms represents a natural step in the communication technology evolution for pharmacists. The ubiquitous nature of SM platforms places them in a prominent position as a medium for relevant pharmacy professional information dissemination, as demonstrated in this study. The increased access to information and the consequent knowledge expansion can lead not only to professional development, but also to better services and patient care.

As one of the major open sources on the Internet, Wikipedia was highly used by pharmacists during work time to access health and medicines information. As Wikipedia is one of the top results from medical queries in general search engines [27], it is not surprising to see it used commonly. However, most participants did not consider Wikipedia as an authoritative source and several of them voiced concerns about its accuracy and reliability. These concerns matched pharmacy literature restriction recommendations on the use of Wikipedia in both pharmacy education [28] and practice [29]. Nevertheless, it is important to emphasize that other studies have pointed out that Wikipedia entries have a high degree of quality compared to the prestigious Encyclopedia Britannica [30] and can be useful as a reference for health students’ assignments [31].

The high use of Wikipedia found in this study is consistent with a high rate of Wikipedia users among pharmacists previously reported [12]. Wikipedia use among pharmacists might be on the rise since the first rate of Wikipedia use published in 2009 had indicated that only 35% of US pharmacists were using it [6]. Wikipedia was commonly used during work time for initial explorations of less known topics related to health and medicines. This is similar to how physicians have been reported using it to get an overview of unfamiliar topics [32]. It is also substantiated by a review that found Wikipedia was widely used as a reference source by health care professionals in general [33]. The Wikipedia editing process was well known by all participants in our study, in contrast with the 2009 US survey where only a third of Wikipedia users knew how it was edited [6].

User-friendliness and ease of access, as well as the fact that Wikipedia often appears in the top 10 results of searches in general search engines such as Google, are important aspects for consideration in developing approaches for conveying information to pharmacists in all areas of the profession [34]. Pharmacists’ contribution to the “free encyclopedia” seems to be negligible, despite, as our study asserts, being highly accessed by pharmacists. Although there is a call for health care professionals, their societies, patient groups, and institutions to join the effort in improving Wikipedia’s health-related entries [27], it is believed that the community who edits health-related entries is very small and is driven by an intrinsic set of values and beliefs [35]. The rationale underlying collaborative projects like wikis is that the joint effort of many actors leads to a better outcome than any actor could achieve individually [1]. Perhaps the best approach to improve medicine and pharmacy entries on Wikipedia would be via collaboration between academia, health organizations, practitioners, and consumers.

The increased access to up-to-date information provided by SM also led pharmacists to engage in another professional task: sharing information. SM made information sharing easier, reaching a bigger audience. Some very active participants saw the opportunity afforded by SM and launched their own platforms, mainly blogs and Facebook groups. It is important to emphasize that when sharing professional information, participants commonly provided links to original sources of information and tried to post the original source of the material. This significant aspect of information sharing behavior might be caused by the fact that our sample consisted of experienced pharmacists (mean 13.3 years of practice [SD 9.8]) and a higher level of education (many had a post-graduate degree or were getting research degrees).

The use of SM channels to spread health- and pharmacy-related knowledge and spark professional discussions should be considered an additional tool to traditional forms of professional interaction in pharmacy. The SM platforms can be complementary and even increase the effectiveness of these traditional ways. The use of Twitter in conferences is seen as example of how the traditional congregation of peers can have their discussions spread beyond the setting of the conference instantly and at the same time causing an impact on the participants’ perceptions and level of interaction within the conference. This is congruent with the assertion that Twitter use during medical conferences was a medical education application [36]. However, the benefits of such application in pharmacy should not be overestimated. A study about Twitter use during a major US pharmacy conference found that Twitter was used by less than 2% of attendees [37]. It has also been reported that almost half of all tweets were made by a tiny group of participants (0.125%) and only a third of tweets were related to educational sessions. Nevertheless, the reach and impact was not verified.

Although our snowball approach led us to recruit key Twitter users, the use of this microblogging platform was less prominent than other SM applications among our participant cohort. This is congruent with findings from surveys among pharmacy preceptors, which revealed that less than 10% of respondents had a Twitter account and that their use was very limited (ie, majority accessed on a monthly bases) [9]. Similarly, a more recent direct observation study on Twitter indicated that less than 1% of practicing US pharmacists had a Twitter account [11]. Most participants who used Twitter in our study used it primarily for professional reasons. This is in contrast to an earlier observation study that evaluated tweet samples and showed that only 10% used Twitter exclusively or predominantly for professional reasons [11]. This cohort of Twitter users might be very similar to our Twitter participants who also used Twitter to both obtain and disseminate pharmacy-related news, recently published pharmacy articles, and other useful and relevant health-related information. Twitter is a suitable application for this purpose since microblogging is designed to offer real-time updates [19], and participants can access important and relevant information up-to-the minute.

Although the use of SNS for recruiting and hiring employees is a new process with limited research [38], this study has found that this use of SNS was perceived to be very common within the pharmacy sector, playing an important role in the pharmacy job market. This seems to be a very beneficial professional use of SNS by pharmacists. It expanded the reach of pharmacists searching for job opportunities. At the same time, it also favored recruiters, who could access a pool of candidates within a closer network (eg, a local Facebook pharmacists’ group). In other words, SNS amplified the “word of mouth” strategy commonly employed in the recruiting of community pharmacists.

The use of SM in pharmacy education described in this study was very similar to previously published approaches [5]. While the Internet, since its first years, facilitated teaching and learning, the need to master the standard mark-up language (HTML) and related concepts (eg, servers, file transfer protocol [FTP] of files, client-side plug-ins, Java applets for interactivity) served as barriers to its use since they required intricate programing [39]. The advent of SM has revolutionized the use of the Internet as an education tool as it has eliminated the interface barriers and information technology constraints for users to create and share content online.

### Limitations

The findings presented should be viewed in light of certain limitations. First, as an exploratory qualitative study with a sample size of 31 pharmacists, the study findings are not generalizable to the pharmacist population. Second, the results may have been impacted by a self-selection bias, despite the fact that not all participants were active SM users. However, a wide range of views on the topic was provided, which could also be interpreted as a strength since participants with more SM expertise can provide more information and insights. Third, as social media is constantly changing due to its dynamic nature, it is advisable to keep in mind that the results represent the situation only at the time of the study.

### Conclusions

Although personal and social reasons generally were the main drivers to join SM (namely Facebook), participants frequently progressed to using SM for professional activities, with most actively engaged with SM in a professional capacity. Primary activities included accessing and sharing professionally relevant information among peers, intraprofessional networking, and job announcements. Wikipedia, YouTube, and Facebook were the most commonly used platforms. However, few professional contributions to the first two were noted. Pharmacists as experts on medicine could provide a great contribution by taking the lead in adding and editing medicine information accessible to consumers in highly accessed SM platforms such as Wikipedia. As the use of SM tends to increase among the population in general, a corresponding expansion of its application in the health sector and pharmacy may also be probable. Consequently, it is expected that this research might stimulate debate and professional use of social media among practitioners as well as stimulate further research on this topic.

## Appendix

Multimedia Appendix 1 Interview guide.

## Abbreviations

SM: social media
SNS: social networking site
