# Supplementary material for: Professional Use of Social Media by Pharmacists: A Qualitative Study
Source: J Med Internet Res. 2016 Sep 23;18(9):e258. doi: 10.2196/jmir.5702 (PMC5055590; doi:10.2196/jmir.5702)
Supplement: Multimedia Appendix 1 [file jmir_v18i9e258_app1.pdf]

Multimedia Appendix 1

Interview guide

1. Knowledge and understanding of social media*<br>a) What do you understand by social media? What do social media mean for you?<br>b) Please could you give me some examples of social media?
2. Use of social media (in particular professional applications)*<br>a) What social media platforms do you use?<br>b) What kind of activities do you perform on social media in a professional capacity?
3. Perceptions of peers' use of social media*<br>a) How do you perceive other pharmacists using social media?
4. Social media and consumers of health<br>a) What are lay persons' perceptions of using social media for health?<br>b) How do you think laypeople and consumers use social media for health purposes?
5. Impact of social media on pharmacy profession*<br>a) What kind of impact do you think social media has or would have on the pharmacy profession?
6. Awareness and opinions about social media policies and guidelines<br>a) Are you aware of social media guidelines and polices for pharmacists? Which ones are you aware of?<br>b) What is your opinion about social media policies and guidelines for pharmacy?

*Themes emerging from these topics/ questions have been reported in this article
